# Report from a Survey of Parents Regarding the Use of Cannabidiol* (Medicinal cannabis)* in Mexican Children with Refractory Epilepsy

**DOI:** 10.1155/2017/2985729

**Published:** 2017-03-14

**Authors:** Carlos G. Aguirre-Velázquez

**Affiliations:** Instituto Tecnológico de Estudios Superiores de Monterrey, Campus Salud, Avenida Morones Prieto 3000 Pte., Col. Los Doctores, 64710 Monterrey, NL, Mexico

## Abstract

Structured online surveys were used to explore the experiences of the parents of children with refractory epilepsy using medicinal cannabis in Mexico during September 2016. The surveys, which were completed in full, were reviewed, and 53 cases of children aged between 9 months and 18 years were identified. Of these, 43 cases (82%) were from Mexico and 10 (18%) were from Latin American countries. Of the 43 Mexican cases, the diagnoses were as follows: 20 cases (47%) had Lennox-Gastaut syndrome (LGS); 13 cases (30%) had unspecified refractory epilepsy (URE); 8 cases (19%) had West syndrome (WS); 1 case (2%) had Doose syndrome (DS); and 1 case (2%) had Ohtahara syndrome (OS). In total, 47.1% of cases had previously been treated with 9 or more anticonvulsant therapies. The parents reported a decrease in convulsions when cannabidiol was used in 81.3% of the cases; a moderate to significant decrease occurred in 51% of cases, and 16% of cases were free from seizure. The number of antiepileptic drugs being used was reduced in 9/43 (20.9%) cases. No serious adverse effects were reported, with only some mild adverse effects, such as increased appetite or changes in sleep patterns, reported in 42% of cases.

## 1. Introduction

Epilepsy is a common and chronic neurological disorder, which frequently requires the use of polypharmacy. It has been calculated that over 50 million people worldwide suffer from epilepsy, with 85% of these patients residing in developing countries such as Mexico. Nearly 2.4 million new cases of epilepsy are diagnosed every year according to the World Health Organization, “Mental health, Neurology, Epilepsy” [1]. About 30–35% of patients have refractory epilepsy (RE), which is defined as a failure to respond to ≥2 antiepileptic drugs according to the new definition of refractoriness proposed by the International League Against Epilepsy (ILAE) Agreement in 2010 [2]. In Mexico, the prevalence rate reported by the Programa Prioritario de Epilepsia [Epilepsy Priority Programme] centers is 11–15/1000 inhabitants [3]. Thus, it is estimated that there are 1.5 million patients with epilepsy in Mexico, with 80% of patients receiving inadequate treatment while 30% of patients have RE. Some studies carried out in Mexico have reported a prevalence of between 1.2 and 3%, with Lennox-Gastaut syndrome presenting in 3% of childhood epilepsy cases [4].

Serious childhood epilepsies are characterized by frequent convulsions, delayed neurological development, and a deterioration in the quality of life of the child, in these cases, which do not respond to conventional treatments. Childhood epilepsies beginning in the first few years of life are frequently characterized by seizures that are resistant to available treatments, including antiepileptic drugs (AEDs), ketogenic diet, high doses of steroids, and surgery [5]. When indicated treatments fail to control their child's seizures, some parents turn to alternative treatments. One of these alternative treatments is cannabidiol-enriched cannabis. The cannabis plant contains approximately 80 cannabinoids, of which cannabidiol and Δ9-tetrahydrocannabinol (THC) are the two most abundant [6, 7]. Families often look for alternative treatments to bridge the gap between the need for effective treatments and the adverse effects and high costs of conventional treatments. Therefore, these patients receive multiple combinations of various antiepileptic drugs; for many of these patients there are no beneficial treatment options.

Over the last decade, interest has emerged in the use of* Cannabis sativa* in home-made products to treat various types of childhood epilepsy, and anecdotal evidence of its success has spread via digital media according to Gupta [8]. From the international point of view, Epilepsy of the ILAE dedicated part of June issue 2014 to the theme “Cannabis and Medical Marijuana for Epilepsy,” which is a support for those who are “interested in working on the subject,” according to Mathern et al. [9].

Gradually medical studies regarding the use of these products have also been published. Gloss and Vickrey [10] conducted a systematic review of four articles reporting on the use of* C. sativa* and concluded that studies in animals have provided sufficient justification for testing in humans; however, there is still no consistent evidence regarding its effectiveness and safety. Porter and Jacobson [11] carried out an observational study of 19 children with RE aged between 2 and 16 years in Stanford University via a survey using social media. The results of that survey showed that 16 out of 19 parents (84%) reported a reduced frequency of seizures with* C. sativa*. Other effects reported were as follows: improvement in mood (79%), increased state of alertness (74%), improved sleep (68%), and a reduction in self-stimulating behaviors (32%). The only adverse effects reported were somnolence (37%) and fatigue (16%). In another survey of 117 parents of children with RE, due to Lennox-Gastaut and infantile spasms, Hussain et al. [12] reported similar improvements over an average treatment time of 6.8 months using an average dose of 4.3 mg/kg/day of cannabidiol (CBD). Devinsky et al. [13] recently published the results of an open multicenter prospective clinical trial, approved by the FDA, using a pharmaceutical product (Epidiolex®) which is cannabinoid based, that is, 99% CBD. There was an average reduction in motor seizures of 36.5% in the 162 children studied, and an adequate safety profile for CBD was noted.

A bioethics review was recently carried into the use of CBD in the Epilepsy Clinic of the Hospital General de México, CDMX [Mexico General Hospital]. This gave rise to three proposals regarding its position and offered important points for consideration in the well-practiced debate on the legalization of cannabis use. One of the proposals suggests that, from a neuroethical point of view, the use of CBD is justified in cases of RE or catastrophic cases, based on international laws safeguarding human life and dignity (Kalkach-Aparicio et al. [14]).

We present the first observational study on the medicinal use of CBD (Medicinal cannabis) in children with RE in Mexico.

## 2. Materials and Methods

An online survey was used to explore the experience of parents administering CBD to children with RE in Mexico. To this end, an online commercial software program was used (http://www.e-encuesta.com) and a license was obtained which provided options for designing an unlimited number of questions of different types (open, closed, and multiple choice), as well as using filters in a packet of up to 5000 e-mail invitations. The system gathered the responses and automatically produced a report using basic statistical analyses. The information was securely stored according to the guidelines of* Amazon Web Services®*.

A survey was designed which consisted of 31 questions in 9 sections. Informed consent was provided and a confidentiality clause was included for personal details, which required a digital signature from the father or mother as means of acceptance. The sections were as follows: personal details, time of evolution and neurological diagnoses, previous number of conventional anticonvulsants, antiepileptic drugs prior to CBD, total number of seizures in the month preceding CBD treatment, CBD product, dose and time of treatment, total number of seizures in the month following CBD treatment, changes in emotional, cognitive, sleep, and dietary state, side-effects observed during treatment with CBD, and open feedback on the use of CBD.

The survey was available for the month of September 2016 and was presented to parents of patients by a link sent via e-mail or the Facebook group porGRACE (porGRACE Association A.C.), which was set up to share information on the use of medicinal cannabis to treat convulsions in children. Participation in the survey was voluntary and via self-selection. The criteria for invitation were as follows: (1) diagnosis of RE, such as Lennox-Gastaut, Dravet, Doose, or other causes, without symptoms being controlled, and (2) current use of medicinal cannabis (cannabidiol alone or in combination). An automated report of all of the information was obtained, which removed surveys of people over the age of 18, partially answered surveys, or surveys of individuals who did not meet the ILAE RE criteria. All completed surveys were printed and individually analyzed. Microsoft Excel 2013® was used to input data and generate graphs.

## 3. Results

8,757 entries were recorded from 53 completed surveys: 43 cases from Mexico and 10 from other Latin American countries. The general characteristics of the 43 Mexican children are shown in Table 1.

The most commonly used product was RSHO-X® Cannabidiol (76.6%), which contains less than 0.1% THC. Its import has recently been approved by COFEPRIS (government institution equivalent to the US FDA). 11.5% of individuals use some sort of combination therapy of CBD + THC (Charlotte's web®) and 11.6% of individuals use different home-made cannabis extract products containing undefined concentrations of cannabinoids.

The results indicate a decrease in convulsions in 81.3% of cases, with 7 cases (16%) free from seizure, 22 cases (51%) showing moderate to significant improvement, and 7 cases (16%) showing slight improvement. No change was seen in 5 cases (11.6%), and 2 cases (4.6%) showed an exacerbation of seizures (Table 2).

**Figure 1:**
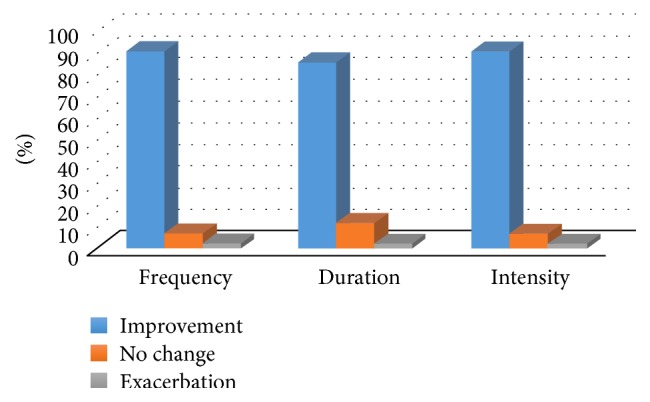
Effect of treatment with CBD on convulsive seizures.

The improvements reported by parents were related not only to the frequency of the seizures, but also to their duration and intensity (Figure 1).

**Figure 2:**
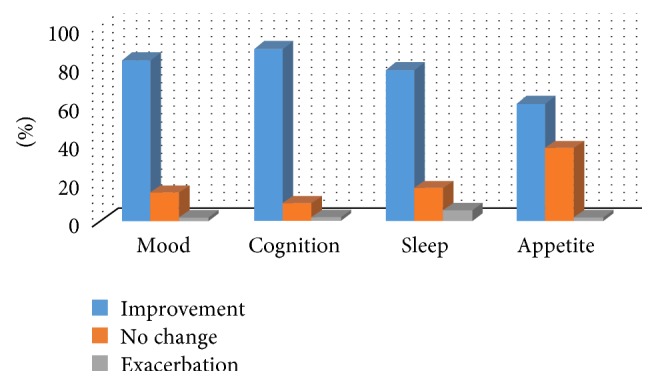
Effects of cannabidiol on quality of life factors.

Benefits reported by parents in relation to quality of life indicators for their children were, for example, improvements in emotional state, attention, communication, sleep patterns, and diet (Figure 2).

No serious adverse effects were reported. In 16 cases (42%) some mild adverse effects were reported, such as increased appetite or changes in sleep patterns. No adverse effects were reported in 27 cases (62.7%; Table 3).

## 4. Discussion

We believe that the reports from parents who participated in our survey are encouraging when it comes to the use of cannabidiol in RE in children. The limitations of this survey are the self-selected cases and the placebo effect, observed as 20% in controlled studies with placebo.

The effectiveness and safety profile of CBD does not vary greatly from what has been reported by other authors such as Devinsky et al. [13] and Porter and Jacobson [11], who report freedom from seizure in 14.5% and 11% of cases, respectively, compared to 16% of cases in our survey. Reductions in the frequency of seizures of over 50%, which is an indicator of the effectiveness of an antiepileptic drug, have been reported by Devinsky et al. (67%) and Porter and Jacobson (42%) compared to the 51% reduction noted in our survey.

With regard to the adverse effects of CBD, these are similar to those reported in the literature [10–13], with the exception of fatigue. This is likely due to the limitations in communication in our cases. While some cases experienced an increase in convulsive seizures during CBD treatment, no epileptic state or sudden unexpected death was reported in the Mexican cases.

Rivera-Olmos and Parra-Bernal in a recent review article [15] commented that preliminary data exist that the use of CBD in extremely rare childhood epilepsies refractory may have a therapeutic effect in intractable crises, but controlled studies based in evidence are still ongoing and have not been completed. The recommendation is that the use of CBD in these cases is within a framework of study where the actual efficiency can be determined and security aspects are monitored.

These results, and published from other in a large number of patients, are encouraging and have revived interest in testing these treatment options, despite the legal restrictions that exist in many countries.

## 5. Conclusions

This is the first observational study carried out in Mexico exploring the use of medicinal cannabis in pediatric RE.

The results of this study indicate that, from the viewpoint of the parents of children with RE, medicinal cannabis is useful as an “add on” treatment for their children since it induces a significant reduction in the frequency, duration, and intensity of the seizures. It also improved aspects of the patients' quality of life in terms of their emotional and cognitive states, their sleep patterns, and their diet. There was an absence of serious adverse effects, with only some tolerable mild adverse effects experienced with this CBD-based treatment.

We believe that these results support the clinical protocols being established in large centers where there are a high number of epilepsy cases and in epilepsy clinics in our country. The actual role of CBD in particular, and medicinal cannabis in general, in epilepsy and other neurological pathologies still needs to be determined.

## Figures and Tables

**Table 1:** General characteristics of 43 Mexican children with RE receiving treatment with CBD.

	Surveys	#/43 (100%)
Age	Minimum 9.5 months	Average: 7.6 yrs ± 4.3
	Maximum 18 years	

Sex	Male	27 (62.7%)
	Female	16 (37.2%)

Evolution	<2 years	8 (18.6%)
	2–6 years	16 (37.2%)
	6–10 years	11 (25.5%)
	>10 years	8 (18.6%)

Diagnoses	Lennox-Gastaut S.	20 (47%)
	West S.	8 (19%)
	Doose S.	1 (2%)
	Ohtahara S.	1 (2%)
	Unspecific R.E.	13 (30%)

# antiepileptic drugs before CBD	1-2	3 (7.5%)
	3-4	25 (62.5%)
	5-6	11 (27.5%)
	7–10	2 (5.0%)
	>10	0 (0.0%)

# antiepileptic drugs After CBD	1-2	14 (32.5%)
	3-4	24 (55.8%)
	5-6	4 (9.3%)
	7–10	0 (0.0%)
	>10	1 (2.3%)

CBD product	CBD 5000 mg	31 (72%)
	CBD 1000 mg	2 (4.6%)
	CBD + THC 5000 mg	3 (6.9%)
	CBD + THC 500 mg	2 (4.6%)
	Other CBD/THC	5 (11.6%)

CBD dose mg/kg/day	<1	3 (6.9%)
	1–3.9	27 (55.2%)
	4–6.9	5 (19.4%)
	7–8.9	3 (6.9%)
	9–11	0 (0.0%)
	Unknown	5 (11.6%)

CBD treatment time in months	<1	4 (9.3%)
	1-2	7 (16.2%)
	2-3	7 (16.2%)
	3-4	12 (27.9%)
	5-6	7 (16.2%)
	6–12	6 (13.9%)
	>12	0 (0%)

**Table 2:** Responses regarding the monthly frequency of seizures occurring due to the use of CBD.

Categories of improvement	% reduction	#/43	% cases
	General	35	81.3%
Free from seizure	100%	7	16%
Significant	80–100%	11	25.5%
Moderate	50–80%	11	25.5%
Slight	25–50%	7	16%
Unchanged	±25%	5	11.6%
Worsening	>25%	2	4.6%

**Table 3:** Adverse effects during treatment with CBD.

Adverse effects^*∗*^	#/43	%
Severe	0	0%
Mild	16	37.2%
Increased appetite	10	
Decreased appetite	3	
Broken sleep	3	
Insomnia	1	
Constipation	2	
Flatulence	2	
Diarrhea	1	
Tics	2	
Increase in seizures	1	
None	27	62.79%

^*∗*^Some cases presented with several adverse effects.
